# Guidance on the Use of Best Available Science under the U.S. Endangered Species Act

**DOI:** 10.1007/s00267-016-0697-z

**Published:** 2016-04-16

**Authors:** Dennis D. Murphy, Paul S. Weiland

**Affiliations:** Biology Department, University of Nevada, Reno, NV 89511 USA; Nossaman LLP, 18101 Von Karman Avenue, Suite 1800, Irvine, CA 92612 USA

**Keywords:** Federal Endangered Species Act, Best available science, Management hypotheses, Effects analysis, Adaptive management

## Abstract

The Endangered Species Act’s best available science mandate has been widely emulated and reflects a Congressional directive to ensure that decisions made under the Act are informed by reliable knowledge applied using a structured approach. We build on a standing literature by describing the role of the best science directive in the Act’s implementation and best practices that can be employed to realize the directive. Next we describe recurring impediments to realizing determinations by the federal wildlife agencies that are based on the best available science. We then identify the types of data, analyses, and modeling efforts that can serve as best science. Finally, we consider the role and application of best available science in effects analysis and adaptive management. We contend that more rigorous adherence by the wildlife agencies to the best available science directive and more assiduous judicial oversight of agency determinations and actions is essential for effective implementation of the Act, particularly where it has substantial ramifications for listed species, stakeholder segments of society, or both.

## Introduction

The Endangered Species Conservation Act of 1969 (ESCA) has been relegated to a footnote in the history of the modern environmental movement in the United States largely due to the passage of its successor, the Endangered Species Act of 1973 (ESA). The latter legislation added regulatory teeth to a statutory scheme to conserve threatened and endangered species and the ecosystems that support them, thereby it gained its notoriety as the “pitbull” of environmental laws (see Quarles 1998). But one provision of the earlier Act, which required the Secretary of the Interior to make determinations whether to list and protect animal and plant species “based on the best scientific and commercial data available,” has been widely emulated and codified in the ESA, other federal wildlife laws, federal pollution control laws, and a diversity of state laws. Although that “best available science” mandate has been a requirement in federal efforts to protect imperiled species for more than four decades, and scientific advances during that period have greatly increased our understanding of such species and their habitats, it commonly is alleged that the federal wildlife agencies charged with administration of the ESA fail to meet that standard (e.g., Hastings et al. 2014; Corn et al. 2013; Holland 2008). A growing body of caselaw and commentary documents that failure (for example, Tucson Herpetological Soc. v. Salazar, 566 F.3d 870 (9th Cir. 2009)); however, absent a definition of best science from Congress and no consistent application of science in determinations by the agencies, the federal courts have proven reluctant to intervene to push those who implement the Act to meet the statute’s stated intent.

As dozens of lawsuits generated by both environmental organizations and regulated communities attest, the U.S. Fish and Wildlife Service (FWS) and National Marine Fisheries Service (NMFS) default repeatedly to surmise and prescribe conservation actions from supposition, even where reliable data are available and targeted analyses offer the opportunity for directed management based on reliable knowledge (Murphy and Weiland 2011). The FWS has acknowledged as much, recently identifying a number of cases wherein federal courts pointed to shortcomings in individual determinations, indicating little effort by the agency to meet prevailing scientific standards and its default to conservation planning by proxy (Murphy and Weiland 2014a). But it is the more pervasive reluctance within the judiciary to consistently require that the agencies meet any technical standard at all that has contributed the sustained unwillingness of the wildlife agencies to meet the legal requirement. Time and again courts have deferred to those agencies’ judgment, even in situations where the absence of scientific support for agency findings is egregious (Fischman and Ruhl 2016). For just one example, *Alaska v. Lubchenco*, Case Nos. 10-271, 11-001, 11-004 (D. Ak. Jan. 19, 2012), aff’d 723 F.3d 1043 (9th Cir. 2013), is a case in which the plaintiff challenged a biological opinion imposing restrictions on harvest from the Atka mackerel and Pacific cod fisheries in the Bering Sea and Aleutian Islands to protect the western distinct population segment of the Stellar sea lion (*Eumetopias jubatus*). NMFS based its determination that harvest of the fish would likely jeopardize the continued existence of the Stellar sea lion, in part, on the basis of the so-called nutritional stress theory. The state of Alaska argued that 13 of the 14 indicators of nutritional stress specified by NMFS showed have no evidence of such stress; in response, the court stated it is not the court’s place “to supplant NMFS’s scientific judgment with its own regarding of what a simple tally of the number of factors weighed for or against its determination might indicate.” Declaring that the federal courts must accord “the highest deference” to agency determinations, the deciding court stated that “although agencies may not act based on pure speculation,” little more is required. Surely Congress did not intend for the best available science directive to be met by a level of analysis that merely exceeds pure speculation. But the common practice in agency determinations often falls short of rigorously engaging available information in any sort of deductive framework (Joly et al. 2010; Murphy and Weiland 2011), and the courts frequently are unwilling to require the wildlife agencies to meet Congress’s clear directive.

The need for a roadmap to assist FWS and NMFS in meeting the best available science standard was recently reinforced by a pair of decisions issued by a federal appeals court—S*an Luis & Delta Mendota Water Auth. v. Jewell*, 747 F.3d 581 (9th Cir. 2014) and *San Luis & Delta Mendota Water Auth. v. Locke*, 776 F.3d 971 (9th Cir. 2014)—involving challenges to biological opinions authorizing the continuing operations of the Central Valley and State Water projects. These projects provide water to more than 20 million Californians and have impacts on the federally protected delta smelt (*Hypomesus transpacificus*) and a number of other listed fish, including Central Valley steelhead (*Oncorhynchus mykiss*) and Sacramento River winter-run Chinook salmon (*Oncorhynchus tshawytscha*), which out-migrate to the Pacific Ocean through the Sacramento-San Joaquin Delta (NMFS 2009). In both cases, a district court considered input from court-appointed technical experts and expert declarants in finding that the FWS and NMFS had not appropriately used available survey data, and sent the agencies back to reanalyze that information and re-craft a conservation action plan. The FWS had determined that when water exports exceed a certain threshold, the rate of salvage (a proxy for mortality) of the threatened delta smelt increases, therefore, water exports “jeopardize the continued existence” of the species. The lower court rejected the agency’s conclusion explaining that the “use of raw salvage data, as opposed to salvage data scaled to population size, is problematic because salvage figures do not account for the size (or relative size) of the smelt population.” But an appeals court reversed the lower court’s corrective finding while acknowledging that the FWS could have better assessed the effects of water exports on delta smelt by normalizing the data to reflect the varying size of the delta smelt population as recommended by a commissioned peer review. The court of appeals effectively concluded that the judiciary is not capable of taking a hard look at even elementary scientific arguments on their merits, and seems to have found that any justification for an agency determination should suffice, no matter how unsubstantial.

We contend that the above decisions and numerous others over the past two decades are inconsistent with Congressional intent and the plain language of the ESA. They demonstrate the reticence of the courts to evaluate agency adherence to the best available science directive, which may well reflect the appreciation of individual judges that they themselves do not have a grasp of science. However, if the wildlife agencies that administer the ESA only aim to meet the standard required by the courts, it is inevitable that their determinations and decisions will be ineffective in conserving species that are protected under the Act and will impose costs on regulated communities that very well may not be warranted.

Given the recent court decisions and burgeoning numbers of resource management conflicts, it is a useful time to revisit the best available science directive in the context of the ESA. Here we examine the regulatory and legal concepts of best available science. We identify ESA decisions that must meet the best science available standard, the tools, analyses, and modeling efforts that characterize science in support of conservation planning, and the common impediments to decision making based on scientific evidence. Thereby we observe how best science can better inform implementation of the Act’s provisions. We also consider how the application of best available science to effects analysis and adaptive management can assist the agencies in meeting the statutory standard.

## Regulatory and Legal Concepts of Best Available Science

The ESA as amended does not impose a single requirement to use best available science. Rather, it promulgates a number of distinct requirements that use similar, but not identical, language. Section 4(b)(1)(A) states the federal wildlife agencies must make listing and delisting decisions “on the basis of the best scientific and commercial data available.” Section 4(b)(2) specifies that federal wildlife agencies “shall designate critical habitat, and make revisions thereto, under subsection (a)(3) on the basis of the best scientific data available and after taking into consideration the economic impact, the impact on national security, and any other relevant impact, of specifying any particular area as critical habitat.” And the interagency consultation provisions of the ESA in section 7(a)(2) state that “[i]n fulfilling the requirements of this paragraph, each agency shall use the best scientific and commercial data available.”

Other federal statutes contain similar language with similar intent. For example, the Marine Mammal Protection Act, enacted in 1972, requires the Secretary of Commerce to prescribe regulations that govern the taking and importing of marine mammals “on the basis of the best scientific evidence available” (MMPA 1972). The Magnuson-Stevens Fishery Conservation Act, enacted in 1976, states that conservation and management measures in fishery management plans “shall be based upon the best scientific information available” (MSFCA 1976). These various provisions all are the apparent progeny of the ESCA; however, there is no legislative history that might further inform our understanding of the requirement.

Both legal scholars and scientists have consolidated these varying requirements for best science into a de facto best available science standard and opined on its intent, scope, and efficacy (for example, Green and Garmestani 2012; Joly et al. 2010; Sullivan et al. 2006; Ruhl 2004; Doremus 2004; Brennan et al. 2002; Smallwood et al. 1999; Carroll et al. 1996; Bogert 1994). A plausible explanation for the genesis of the best available science requirement was offered by Doremus (2004) who opined that “the best available science mandate was generally intended to ensure objective, value-neutral decision making by specially trained experts.” She acknowledged the fact that implementation of the ESA often requires reconciling competing values, assuring that science is unlikely to be the exclusive determinant of agency decisions. We agree that reconciliation of technical disputes using expert judgment may be an important application of the best available science directive, and “weight-of-evidence” approaches may be a reasonable default to inform some agency decisions. But the essential value in the directive is the implicit acknowledgment that application of the scientific method should be the standard in gathering and assessing information that is applied to conservation decisions and actions under the ESA. Moreover, the conceptual framework and approach of the scientific method “provides grounds for establishing a process that can serve to parse out technical or scientific issues from policy considerations” (Murphy and Weiland 2011).

Several treatments have contributed general guidance regarding application of the best available science standard. For example, Doremus (2004) suggested that decisions under the ESA should meet the needs of species as revealed by scientific data and should only restrict human activity to the extent required for the protection of such species. But such general counsel has proven insufficient to guide the wildlife agencies toward scientifically defensible determinations, to inform the regulated community of the basis in reliable knowledge for agency decisions, and to assist reviewing courts in implementing and assessing application of the best available science standard. Given the sheer number of federal cases regarding listed species and agency directives intended to conserve them, we think that more refined guidance is needed. At the same time, to the extent such guidance is overly prescriptive, it may not fare well as scientific advances are made. For example, although at the time of enactment of the ESA, biologists generally considered the “balance of nature” concept to be credible; that theory has since been discredited. Theories about ecological phenomena, such as inter-specific competition, predator-prey interactions, and density dependence have been reinterpreted. Thus, there is a trade-off between technical guidance that is too general to provide a meaningful standard against which agency determinations can be evaluated, and guidance that is closely tied to prevailing hypotheses and theories that ultimately may be refined, transformed, or abandoned altogether.

The National Research Council (NRC) offered the wildlife agencies advice in a committee report on the best available science standard in fisheries management (NRC 2004). The NRC identified six criteria for ensuring use of the best available scientific information in fisheries management—relevance, inclusiveness, objectivity, transparency and openness, timeliness, and peer review. Those criteria are consistent with information quality guidelines and the information quality bulletin for peer review developed by the Office of Management and Budget in response to enactment of the Information Quality Act (OMB 2002, 2004); but predictably they provide only de minimis direction to those who craft determinations for the wildlife agencies. For example, the NRC opined that “[d]ata collection and analysis should be unbiased and obtained from credible sources” to meet the objectivity criterion (NRC 2004). But, the NRC did not specify standards or describe an explicit process by which the agencies might ascertain whether and to what extent a data set may be representative of pertinent environmental phenomena so as to warrant application in management planning, or whether a particular analysis might provide an objective empirical basis for inferences that can be applied in identifying effective management actions.

By way of comparison, the American Fisheries Society (AFS) prescribed a set of common elements of studies or analyses that are necessary to, in their words, “achieve high quality science” (AFS 2006). The AFS contended that high-quality science includes “a clear statement of objectives; a conceptual model; a good experimental design and standardized method for collecting data; statistical rigor and sound logic; clear documentation of methods, results, and conclusions; and peer review.” Whereas the NRC referenced objectivity and relevance, and focused on the quality of data and analyses that agencies rely upon in making decisions, the AFS emphasized properly interpreting and synthesizing data and analyses, and using them in identifying and selecting from among resource management options. Joly et al. (2010) referred to the latter activities as addressing whether data and analyses under consideration are in fact “scientific” in application. Yet no attempts have been made to identify the requisite information and technical methods that must be used to support an agency determination, so that it can be judged as meeting the “best available science” directive.

## Best Available Science: Science Background

Science is a process. It is not a product or the outcome of deliberations. In that light, the best available science directive rightfully references, not science, but “scientific data,” meaning an element or product of the scientific process or a synthesis of the most reliable knowledge at a point in time. Science “consists of confronting different descriptions of how the world works with data, using data to arbitrate between different descriptions, and using the ‘best’ descriptions to make additional predictions or decisions” (Hilborn and Mangel 1997). The principal elements of the scientific method are formulation of hypotheses regarding the relation between two or more variables, collecting and analyzing data on those relations, interpreting the results, and formulating new hypotheses. Richard Feynman (1974) famously differentiated science from what he referenced as “cargo cult science,” which does not follow the scientific method. Instead, the hallmark of that lesser science is unwillingness to question one’s own theories, hypotheses, and results, including reticence to investigate alternative explanations for the phenomena being studied. Determinations by wildlife agencies that are intended to provide for the survival and recovery of listed species are frequently made by assertion, not deduction (for example, Longcore et al. 2007), therefore do not qualify as science.

It is widely accepted that findings from rigorous empirical research that uses the scientific method provide for a more reliable understanding of natural systems than do alternative means of reasoning to reach conclusions. Research or monitoring carried out in an experimental framework, enjoying the benefits of control and replication, is the preferred means of confronting and resolving the breadth of uncertainties that limit the ability of resource managers to design and implement effective conservation actions (see Table 1, but consider McGarvey 2007). Where demographic data are available in time series, that information should be critically evaluated then applied in numerical models that link environmental stressor data to species and habitat responses to test management action scenarios. Where data that are derived from surveys approached in an experimental framework are not available, Bayesian probability approaches can make use of observational information. If the approaches taken are reasoned and deductive, agency determinations can meet the best available science, even if they use inferences from the same species from other geographic areas or inferences from valid surrogate species in the same or similar circumstances. Best available science also can emerge from expert elicitation, as long as it is not used as a substitute for pertinent existing data, analyses, or model applications (Martin et al. 2012, McBride and Burgman 2012).

**Table 1:** Four categories of uncertainties encountered in effects analyses and implementation of adaptive management (derived from Buneau et al. 2015)

Environmental variability	The probability of many environmental phenomena, including episodic events such as wildfires and earthquakes, near-term weather extremes, and future climate, is uncertain. Drivers of habitat extent and quality, such as flow levels in river systems, annual variability in the phenologies of growing seasons, the distribution of temperature maxima and precipitation, and the presence and abundance of predators and prey, are prime determinants of the distribution and population dynamics of species. Yet these sources of uncertainty are largely irreducible. Advances in modeling and expanded time-series data sets can lead to better estimates of the likely distribution of future conditions and target species responses
Structural uncertainty	Although the fundamental relations between physical conditions at landscape and smaller extents, habitat quantity and quality, and reproductive success sometimes can be inferred from available data, uncertainties inevitably remain concerning the functional form of some relations. What aspects of landscape condition vary in what spatial and temporal patterns to affect habitat extent and quality, how does habitat condition affect local population and metapopulation dynamics. Structural uncertainty can be reduced through research, monitoring, and improvements to models
Parametric uncertainty	Even where the structure of ecological relationships is well known, uncertainty can remain as to the strength of those relationships. For example, what amount of habitat for an imperiled shorebird is available at a given river stage, what minimal abundance of a rare plant is required to support its pollinators, and what salinity level is tolerated by an estuarine fish at each life stage. As with structural uncertainty, those uncertainties can be reduced through research and monitoring and incorporated into models; however, varying over time and by location they can resist resolution
Observation uncertainty	Neither estimates of population size and reproduction, nor habitat structure and composition can be fully accurate. Degrees of error and direction of bias can vary with species characteristics, habitat attributes, and level of sampling effort, thus differ across both space and time. Rigorous design and level of effort in a monitoring program can reduce observation error and, in some designs, estimate the error in targeted surveys, which allows for more accuracy the resulting information

Data deficiencies frequently dominate in conservation planning, particularly where species are elusive and limited, or where haphazard investigations or assessments have taken place.^1^ Furthermore, where listed species have been the targets of research, seldom have hypotheses with direct management relevance been confronted with data pertinent to management. These facts both complicate and at the same time highlight the need for efforts to employ structured decision making and rigorous techniques. In practice, agency determinations are often made on the basis of observations of a species or its habitat, or a proxy for a species, such as land-cover type, or based on a predicted (but usually untested) relationship between the impacts of an activity and the response of a species, its populations, or its habitats. The wildlife agencies rarely, if ever, have the data and analyses they believe are sufficient to make fully informed decisions regarding at-risk species in natural systems. Nonetheless, they frequently have data and analyses that can inform decisions, provided the agencies critically evaluate those data and analyses in context, using available modeling tools, and recognizing the limits of inferences that can be drawn from the available information (Murphy 1990).

A conservation plan or agency determination can benefit from the results of research that employ the scientific method and other technical information, which can vary dramatically in quality and utility. It is usually necessary for the wildlife agencies to draw from a wide range of scientific disciplines, including molecular, population, and theoretical genetics and physiological, behavioral, population, community, and landscape ecology (also see National Research Council 1995, Ruckelshaus and Darm 2006). All must be applied in a spatially and temporally explicit context, and informed by contemporary analytical methods. Demographic modeling, usually taking the form of population viability analysis (PVA), is the de rigueur tool used “to assess threats to a species’ persistence, and to intervene before declines become irreversible” (Noon et al. 1999). PVA, whether informed by minimalist data sets or long time-series census data, and subject to appropriate sensitivity analysis, is a critical component of defensible management decisions for listed species. The results of demographic modeling must be integrated into any assessment of the effects of environmental stressors on a listed species or appraisal of how an ongoing or proposed action is likely to affect the likelihood of survival of a species over a given time frame (Murphy and Weiland 2011).

The predictive value of population viability analyses is proportional to the extent and robustness of available data, and is only useful when there is adequate information regarding the demographic status and trends of a listed species and the extent and quality of its habitats. Where available information is not adequate, the assumptions that must be made in order to undertake PVA and the uncertainty associated with the results from the PVA may lead to the conclusion that the potential benefits of conducting a PVA are outweighed by its costs (in terms of time and resources) and potential for policy makers and the public to overlook uncertainty. Rather than simply default to ad hoc best professional judgment in such circumstances, resource managers are obliged to look to alternative science-based tools, such as weight-of-evidence approaches, model selection, or structured expert elicitation (Johnson and Onland 2004; Runge et al. 2011).

## Decisions that are Subject to Best Available Science

The purpose of the Endangered Species Act is to conserve threatened and endangered species and the ecosystems upon which they depend. That overarching goal is realized foremost by prohibitions on the “take” of listed species, but also by other means including the consultation provisions applicable to actions of the federal government (ESA 1973, as amended).^2^ The critical regulatory provisions of the ESA are included in sections 4, 7, and 10 of the Act. These include the processes for listing and delisting species, designation, and revision of a critical habitat designation, interagency consultation, and conservation planning. The process for listing and delisting species as either threatened or endangered is set forth in section 4(a)(1). Once a species is listed as endangered, it benefits from a number of protections, including those set forth in section 9 of the ESA, which can be extended to species listed as threatened. Section 4(b)(1)(A) requires the federal wildlife agencies to meet the best available science standard when making listing and delisting decisions. Section 4(a)(3) sets forth the process of designating and revising critical habitat. If an area is designated as critical habitat, then a federal agency must complete interagency consultation before authorizing, funding, or carrying out any action that may affect that habitat (see below). Section 4(b)(2) requires the federal wildlife agencies to meet the best available science standard when designating or making revisions to critical habitat.

The interagency consultation provisions of the ESA are in section 7(a)(2); they require all federal agencies, in consultation with and with the assistance of the federal wildlife agencies, to insure that any action authorized, funded, or carried out by such agency is not likely to jeopardize the continued existence of any listed species or result in the destruction or adverse modification of the critical habitat of such species (USFWS and NMFS 1998). The elements of the consultation process include preparation of a biological assessment by the federal agency proposing an action, a biological opinion regarding the effects of the action by the relevant federal wildlife agencies, and, commonly, an incidental take statement (which authorizes take of a listed species incidental to the action subject to consultation). The interagency consultation provisions also require the federal wildlife agencies to meet the best available science standard.

The conservation planning provisions of the ESA are in section 10(a), and they apply where persons or entities other than federal agencies seek to take species that are protected under section 9 of the ESA. There is no express requirement that the federal wildlife agencies meet the best available science standard in reviewing habitat conservation plans and issuing incidental take permits under section 10(a). However, because such actions are subject to the interagency consultation provisions of the ESA, they must comply with the best available science directive through the consultation process. In each case, the text of the ESA clarifies that it is not sufficient for agencies to simply compile available data. Rather, the agencies have a duty to make decisions “on the basis of,” that is, “to use” the best available data. To do so, the agencies must critically assess existing data and thereby identify reliable knowledge, then integrate those data and analyses into models that link species performance with environmental stressor effects, and then evaluate the results in light of the relevant decision-making obligation. These steps mirror myriad step-wise decision-making processes in environmental policy (Murphy and Weiland 2011; NRC 2011, 2009; U.S. EPA 2003).

The statutory criteria in and regulatory criteria associated with section 4 of the ESA must be met and informed by ecological and genetic information. A reasonable expectation of listing and delisting determinations, critical habitat designations, and recovery planning is that the wildlife agencies will use reliable observations, data, and inferences to establish the taxonomic status and ecology of a species proposed for listing, and the demographic status and trend and geographic extent and condition of the habitat of a listed species. Necessary data to meet section 4 criteria include those that bear on taxonomic and demographic distinctness, which are used to inform the legitimacy of a listing decision; data on the distribution and abundance of a species, which are essential to informing a listing decision and from which a species’ status and trends can be inferred; and data that characterize resource use by the species, define its habitat, and identify environmental stressors to the species, which are used to inform actions that can contribute to species recovery and, ultimately, delisting (Table 2).

**Table 2:** Statutory and regulatory criteria for determining whether a species warrants listing, designation of critical habitat, and setting recovery targets, and the types of data that may be used to meet those criteria

Agency determinations under sections 4, 7, and 10 of the Act may fairly be judged as having used the best available scientific information when the following eight methods and best practices are employed.

The agency determination is based on the results of *tests of explicit management*-*relevant hypotheses*. Such tests use data and observations that are pertinent to and reliable for assessment of alternative management scenarios and methods that are consistent with contemporary theory and analytical practices.

*Analyses areclearly described andconsider all available pertinent information*. They cite previous studies, regardless of whether those studies supported the determination, and explain potential reasons for discrepancies. Information quality or reliability may be differentiated and ranked on the basis of the reliability of its sources; for example, information from (independent) peer-reviewed publications would typically be recognized as superior to information from agency publications that have been subjected only to internal review. Information from those sources would be recognized above that from unpublished, but peer-reviewed reports, and information that has not been subject to review.

*Analyses identify and describe assumptions and uncertainties* in the data used to quantify relationships between the target species, its habitat, and potential environmental stressors. Documentation supporting a determination should describe the limitations of all data and analyses used to inform conservation actions, including the quality and variability of data.

*Conceptual ecological models presented in graphical form* are used to articulate the relationships between the listed species and ecosystem components and processes, including both natural and anthropogenic environmental stressors. The model describes in spatial and temporal context the linkages among population attributes, habitat conditions and other ecosystem attributes, impacts or effects of the action that result in take, and potential responses by the listed species to conservation actions. The conceptual models used to inform the agency determination should reflect the knowledge of professionals representing a wide range of expertise.

The analysis of the effects of actions requiring consultation *employ rigorous specification of response variables and environmental factors that affect habitat extent and quality* and is informed by the conceptual model and additional expert knowledge.

*Life*-*cycle models are used to address temporally relevant aspects* of the relations among the listed species, its habitat, and factors that affect its survival and reproduction, which may vary among seasons, across life stages, and throughout its distributional range.

*Data that are engaged in support of the agency determination are presented in spatially explicit context and format.* Available data on the survival, reproduction, distribution and abundance of the target species, the extent and quality of its habitat, and environmental stressors should be presented on maps whenever possible.

*Population viability analysis*, or demographic models that may use incidence functions or quasi-extinction estimation, are employed to estimate the probability that the listed species or targeted population (or populations) will be sustained under varying stressor effects and management action scenarios.

*Analyses use direct measures whenever possible*. Ideally data on survival and reproduction of the listed species, its habitat, and environmental stressors should be gathered from the area subject to the determination. Information on responses of the listed species or closely related species from other locations may prove useful. Information from surrogate species or use of proxy measures should not be considered unless similarities in the responses of the listed species and surrogate species to salient environmental factors have been thoroughly described and the proxy relationships explicitly validated.

*The analyses supporting the determination provide the basis for the development of monitoring* schema. The results of population viability analyses and other quantitative models inform the selection of response variables and covariates and identification of management triggers and thresholds. There must be institutional capacity to interpret data and adjust management actions on the basis of data and analyses that reflect and use the best available scientific information.

The availability of high-quality technical information does not guarantee scientifically defensible agency decisions and determinations. It is a necessary prerequisite for sound effects analysis, but it is not by itself sufficient. Rather, it must be analyzed rigorously by agency staff who have sufficient time, resources, and training; who are willing to question any hypotheses regarding the responses of the listed species to environmental conditions and management; and who are compelled to pursue alternative explanations for ecological phenomena and consider alternative management scenarios in the development of conservation (management) plans.

## Impediments to Basing Decisions on the Best Science Available

There are a number of potential impediments to the identification, presentation, and application of best available science in implementing the ESA. Perhaps the most significant is the wildlife agencies’ lack of institutional capacity, which frequently results from insufficient staff expertise, insufficient financial resources, or both. Doremus (2008) argued that to improve the scientific integrity of agency decisions, the agencies should “hire scientific analysts with more education, at higher pay grades.” It seems that lack of capacity is inevitable given the chasm that exists between the obligations imposed upon the wildlife agencies in their authorizing legislation and the financial resources provided to those agencies (Clark and McCool 1996; Woody 2011).

The lack of institutional capacity in the wildlife agencies can be compounded by the time limits within which the agencies must make decisions. Under both sections 4 and 7 of the ESA, agencies must comply with prescribed time limits when making decisions about listing, critical habitat, and consultation (for example, consultation must be completed in 90 days). When both capacity and time are insufficient, the likelihood that such decisions will not be based on the best available science increases. Additional impediments, many of which are well known, may arise as a consequence of lack of institutional capacity (Table 3) (Murphy and Weiland 2011).

**Table 3:** Impediments to decision making based on the best available science

Impediment	Type of effect or outcome	Specific example
*Lack of institutional capacity*, including Insufficient agency expertise, insufficient financial resources, or both, contributing to poor quality decision making	• Failure to develop and incorporate a life-cycle model into an agency jeopardy determination • Failure to perform quality control with respect to a data set provided in a listing petition	For example, whereas in a biological opinion analyzing effects of a proposed action on Sacramento River winter-run Chinook salmon shortly after the species was listed identified the need to develop a life-cycle model for the species as a conservation recommendation, for the subsequent two decades the National Marine Fisheries Service failed to develop and apply such a model in part due to a lack of institutional capacity (National Marine Fisheries Service 1993, 2009)
*Incomplete presentation of information* that is relevant to the determination or decision under consideration	• Failure to consider and report on relevant and readily available data, analyses, or conclusions	For example, the Fish and Wildlife Service failed to consider recent survey data that provided evidence of a decline in the relative abundance of delta smelt when preparing a biological opinion (NRDC v. Kempthorne, 506 F. Supp. 2d 322 (E.D. Cal. 2007))
*Misinterpretation of findings* that accompany available research, monitoring, and modeling efforts	• Failure to adequately take into account assumptions that accompany analyses or limitations reported in association with findings	For example, where the Fish and Wildlife Service withdrew a proposed rule to list the flat-tailed horned lizard on the grounds the species is persisting in the vast majority of its range; that was based on a single capture-mark-recapture study that found no evidence of a large decline in population in two discrete sections of the species’ range (Tucson Herpetological Society v. Salazar, 566 F.3d 870 (9th Cir. 2009))
*Misrepresentation of findings* that accompany available research, monitoring, and modeling	• Representing population estimates that are based on sampling within a fraction of a species’ habitat as census data • Use of findings regarding the response of one species to an environmental change or disturbance to draw inference regarding the response of a different species without disclosing and accounting for pertinent differences between the species	For example, the National Marine Fisheries Service used data regarding survival of hatchery Chinook salmon to predict the behavioral responses of steelhead and green sturgeon absent any effort to first ascertain whether the former is an effective surrogate for the latter and despite the substantially different life histories of the two species (Murphy and Weiland 2011)
*Inappropriate emphasis* resulting from the presentation of findings out of full landscape or temporal context, or adequately considering the ecology and behavior of the listed species	• Not interpreting available demographic data using life history information and understanding of environmental stressors • Reporting short-term data on trends in a species’ relative abundance without disclosing available long-term trend data	For example, the California Fish and Game Commission designated the tricolored blackbird a candidate for listing under the State’s Endangered Species Act based on a decline in estimates of the species abundance recorded in surveys completed in 2008, 2011, and 2014, disregarding a dozen other surveys completed during the previous four decades (California Department of Fish and Wildlife 2015)
*Assumption that published conclusions are valid*, resulting from a failure to critically analyze conclusions that are presented as part of an empirical study	• Reliance on a publication regarding the effects of an environmental change or certain types of disturbance on a species, even where other scientific information is inconsistent with the results or inferences reported in the article	For example, in response to an administrative appeal from denial of an Information Quality Act request for correction with respect to certain information in a biological opinion regarding operations of water export projects in California, the Fish and Wildlife Service stated that it “accepts the peer review processes of scientific journals and thus, the scientific validity of the paper’s conclusions” (U.S. Fish and Wildlife Service 2009)
*Use of agency staff as researchers, analysts, and advocates* for agency determinations in regulatory and judicial proceedings	• Use of a staff biologist who had published research directly relevant to the agency decision to evaluate the available scientific information and craft the decision	For example, a science review panel asked to review a proposed rule with respect to the status of the gray wolf on the list of threatened and endangered species noted that the rule relied heavily on an article authored by four Fish and Wildlife Service biologists and accepted the conclusions in the article uncritically (National Center for Ecological Analysis and Synthesis 2014)
*Use of peer review as a substitute for rigorous structured decision*-*making* by agency staff or consultants, who should possess adequate expertise, resources, and time to complete their task	• Using peer review of a completed determination to replace structured effects analysis as the vehicle to identify and incorporate scientific knowledge into an agency decision-making process	For example, the Secretaries of the Interior and Commerce asked the National Research Council to review the draft Bay Delta Conservation Plan in terms of its use of science and adaptive management, and the panel found the Plan to be incomplete or unclear in a variety of attributes and approaches, including due to the absence of an effects analysis (National Research Council 2011)
*Bias* based on prejudice or unreasoned judgment that forecloses objective identification, presentation, and application of data, analyses, and interpretations	• Agency use of staff to evaluate or craft determinations, despite knowing a priori that such personnel are advocates who have decided outcomes before evaluation • Disregarding peer reviews without explanation • Lack of information in a determination regarding uncertainty, data variability, or estimation error	For example, the National Marine Fisheries Service listed the Arctic subspecies of ringed seal—while acknowledging the inference of experts that its population numbers in the millions—based on projections of sea ice loss, but absent data that links projected sea ice loss to a decline in the population (Alaska Oil & Gas Assn v. NMFS, Case No. 14-29 (D. Ak. March 11, 2016))

The use of independent scientific review (peer review) can contribute to more robust and defensible decision documents (Sunstein 2002; Meffe et al. 1998). But reliance on scientific review by disciplinary experts from outside the agencies as the means of integrating best available science into agency determinations is inappropriate. In almost all cases reviewers are poorly compensated or take on review tasks as volunteers, hence operate with more constraints and fewer resources than the agencies whose work they review. Furthermore, it is commonplace for regulatory agencies to eschew the basic prerequisites for meaningful and effective review. Those prerequisites include a reasonable scope of review; sufficient time and resources to conduct a review; reviewer participants selected by agency staff other than those who prepared the documents subject to review; and provision of all pertinent documentation to reviewers, including materials developed within the agency, products from the academic community, and information available from stakeholders (for example, Delta Science Program 2013, see Appendix I).

Bias can be the most pernicious impediment to application of best available science. There are many cases of the introduction of bias into decision-making processes by federal agencies both at the staff level and at the political appointee level when implementing the ESA (Wilhere 2012; Doremus 2008). Bias may be a consequence of purposeful conduct, but it also may be inadvertent (Wilhere 2012). Bias can result in decisions that are more or less protective of targeted or non-targeted species, more or less burdensome for the regulated community, and more or less cost efficient. In many circumstances, it may be infeasible to ascertain whether an identified impediment to the use of best available science results from lack of institutional capacity, or bias, or both.

## Effects Analysis and Adaptive Management

Adaptive management is described by the Department of the Interior as “a decision process that promotes flexible decision making that can be adjusted in the face of uncertainties as outcomes from management actions become better understood. [It] recognizes the importance of natural variability in contributing to ecological resilience and productivity… [and] emphasizes learning while doing as a means to more effective decisions and enhanced benefits” (Williams et al. 2009). Over the past two decades, the wildlife agencies have increasingly leaned on adaptive management (Holling 1978; Walters 1986; Johnson et al. 1993) to address uncertainties associated with implementation and efficacy of management actions prescribed in biological opinions and habitat conservation plans. Green and Garmestani (2012) contend that the best available science standard should encapsulate scientific methods rather than use of science per se, and they argue that adaptive management provides a means “to institute learning infrastructure for iterative endangered species management.” In fact, adaptive management can be a means by which best available science can inform management actions undertaken via interagency consultation under section 7 and conservation planning under section 10. Whereas biological opinions and habitat conservation plans dating to the early 1990s largely eschewed adaptive management, adaptive management is now invoked and required in most biological opinions and habitat conservation plans permitted by the wildlife agencies as mitigation for spatially extensive or long-term effects on listed species. It has become almost de rigueur for the wildlife agencies to invoke adaptive management as a means of resolving uncertainties that should be fully addressed in an agency determination.

Adaptive management aims to reduce the effects of uncertainty on decision making by improving understanding of environmental phenomena and system drivers, the habitat and population dynamics of the listed species, and the effectiveness of management actions. Updating models on the basis of new information obtained through adaptive management improves projection of the outcomes of different management scenarios, quantification of uncertainty, and prioritization of information needs. Adaptive management allows for confrontation of management hypotheses with new information and revision of those hypotheses.

The Department of the Interior’s approach to adaptive management sidesteps discussion of best available science (Williams et al. 2009); however, for adaptive management to be effective, efficient, and accountable it must be supported by a structured decision-making process that is informed by best available science. Murphy and Weiland (2014b) describe two phases of adaptive management, both of which require best available science: the process of making an initial agency determination (which takes the form of an effects analysis in the section 7 context) and the experimental, adaptive implementation of the conservation response or action in the determination. Adaptive management cannot fix assumptions about the ecology or behavior of a listed species or the effects of specific environmental stressors that are fundamentally incorrect, such as whether data on species’ occurrences are sufficient to infer trends in its relative abundance or its responses to actions that require consultation. Nor can adaptive management correct a management action that is ineffective in design or implementation. Adaptive management is not a way of “doing science,” as is suggested by Green and Garmestani (2012); instead, it is an approach to resource management that can contribute to success in meeting the management objectives established in the agency determination. However, the wildlife agencies often neglect key steps in the adaptive management process, and the judiciary only strikes down agency misuse of adaptive management in the most glaring of circumstances (Fischman and Ruhl 2016). Because the decisions made by federal wildlife agencies affect both listed species and stakeholders, sometimes in life-altering and irreversible ways, agency practice should hone more closely to the formal concept of adaptive management, while informing its requisite process steps with best available science.

Murphy and Weiland (2014b) described more than a dozen sequential steps in structured effects analysis and implementation of adaptive management that require scientific expertise, vetting, or independent confirmation. Six categorical activity areas that require or benefit from best available science include:

*Developing and specifying conceptual ecological models for the listed species*. As described above, conceptual models are boxes and arrows diagrams that posit the relations among the listed species and environmental factors and processes that affect them. Those models benefit from open dialog among experts about the nature of the species, its habitat, and threats to one or both, serve to inform formulation of goals and objectives, identification of candidate management strategies, indicators, and monitoring approaches, and identify the sources of variability and uncertainty that influence the success of an adaptive management effort (Fischenich 2008; Fischenich et al. 2012). Conceptual ecological models should be informed by the best available scientific information, particularly rigorous, peer-reviewed empirical research where available, but the discrete relationships they describe frequently vary both because the extent of scientific support for them varies and because the magnitude of the relationship (or signal) varies.

*Harvesting and assessing pertinent and reliable data, analyses, and models*. The search for and identification of pertinent and reliable data on the listed species and its habitat and previous analyses of those data is an essential step in adaptive management that must be undertaken by experts from a range of disciplines. The utility of existing information needs to be considered in light of information gaps. Critical analysis of data, methods, results, and interpretations, and clear identification of their limitations and associated uncertainties, informs analysis of alternative management actions. The validation of proxy measures or surrogate species may be necessary in identifying the best available scientific information and in developing quantitative models and monitoring schemes (see Murphy and Weiland 2014a).

*Developing candidate management actions on the basis of conceptual models, then confronting management alternatives as hypotheses with available data and standing or newly initiated analyses.* Candidate management activities must be treated as hypotheses and iteratively confronted with available data. Such hypotheses are structured to differentiate between environmental stressors that may directly affect the status and trends of the listed species, and those that may simply be correlated with demographic changes (Jacobson et al. 2015). Hypotheses are designed to consider hierarchies of environmental stressors, mechanisms by which management actions affect environmental attributes, variable specification, and the costs and benefits of alternative actions. A management action that is not falsified through the hypothesis testing process, that is, is supported by available data, may be a reasonable candidate for implementation (also see Murphy and Noon 1991, 1992).

*Building quantitative models that investigate the effects of environmental stressors and management actions on the listed species and its habitat.* Quantitative operational models are manifestations of conceptual models; they use population viability analysis or other demographic models to assess the effects of alternative operations and mitigation scenarios on the listed species. The construction of quantitative models requires formulation and parameterization of physical and ecological relations to characterize interactions between species response variables, habitat elements, and environmental stressors; sensitivity analyses contribute to identifying the variables that may have substantial effects on model outputs. Model runs are used to select from among alternative management actions that vary in effectiveness and efficacy in meeting species objectives, costs, and impacts on stakeholders.

*Designing monitoring schemes that can be implemented experimentally.* Monitoring is frequently treated as a rudimentary exercise in counting or measurement. However, monitoring in support of adaptive management demands rigorous design that follows from clear identification of programmatic goals and objectives; identification of reliable, scientifically defensible indicators of meaningful changes in the conditions of the targeted listed species, its resources, and other ecological variables; specification of ranges of values of variables that will trigger changes in management; and collection of data that allows for detection of those changes.

*Use of independent review to interpret monitoring data*. Adaptive management advances by adjusting management actions to make them more effective and efficient over time, and by using inferences from incoming data and analyses to adjust not only the monitoring program, but also conceptual and quantitative models of the system. Decisions about real-time adjustments to data collection must consider whether the spatial and temporal bounds of the monitoring program are well reflected in the distribution of sampling that occurs across space and time. Indicator variables need to be re-evaluated periodically to address their precision and to confirm that they provide salient information regarding the listed species.

## Conclusion

There no doubt is a mismatch between the considerable expectations imposed on the federal wildlife agencies in meeting the objectives of the ESA and the resources provided them to do so. This is compounded by uncertainties regarding listed species, their habitats, and the ecosystems that support them. But too frequently the agencies base formal determinations, decisions, and findings on assertion and surmise, rather than on the best available science. They argue that data limitations and time constraints make it impossible to meet the best available science directive from Congress, and sympathetic courts to give them a free pass. This recurs repeatedly even in those circumstances where agency decisions involve species actually on the brink of extinction, or where agency actions have demonstrated impacts on sensitive sectors or subsectors of the economy. Surely the wildlife agencies are challenged to make defensible determinations, but even where the agencies have access to pertinent scientific information they may use that information in applications beyond which it is valid, selectively use data to support predetermined management responses, and employ proxy measures and surrogates without validation.

We contend that the wildlife agencies must acknowledge and the courts must recognize that there are three primary obstacles to the wildlife agencies meeting Congress’ directive to use best science in implementing the ESA. First, the agencies only infrequently engage in efforts to identify, and then appropriately employ, pertinent data and other reliable knowledge to inform their determinations. To meet the best available science directive, the agencies must systematically and transparently apply structured approaches using conceptual and quantitative models informed by well-vetted data when making determinations under the Act. Second, the agencies frequently reference uncertainties regarding the ecology of a listed species and its environment to validate the arbitrary, selective, and uncritical use of data and technical information. To meet the best science directive, the agencies must use experts to identify reliable and pertinent information then apply it in testing management-relevant hypotheses as means of assessing candidate management actions. Third, the agencies increasingly turn to adaptive management as a *post hoc* means of addressing uncertainty in their determinations. To meet the best science directive, adaptive management should be employed, not to test the effectiveness and efficacy of agency determinations, but to better resolve (fine tune) such determinations using reliable knowledge in a structured process.

Ending the era of blind deference by the federal courts to the wildlife agencies’ prerogative in crafting and arguing for its determinations could well be the greatest possible future contribution to meeting the purposes of the ESA set out in section 2 of the Act and reflected in the best available science mandate. Agency determinations that use structured approaches to analyzing the effects of actions authorized under section 7 and 10 of the ESA, that employ a hypothesis-driven process to identify and implement conservation responses to listing, designation of critical habitat, and recovery planning, and that provide substantive guidance to those implementing mitigatory management in an adaptive framework are determinations that the federal courts can with confidence find to be informed by best available science.

## Footnotes

1 Acknowledging that frequently there are limited data regarding many species that have qualified for or could qualify for listing under the Endangered Species Act, there must be sufficient data regarding the status and trend of the species to trigger listing itself (see Table 2). To list a species, the relevant wildlife agency must determine, solely on the basis of the best scientific and commercial data available that (1) it is in danger of extinction throughout all or a significant portion of its range or (2) it is likely to become in danger of extinction within the foreseeable future throughout all or a significant portion of its range.

2 Section 9 of the Endangered Species Act prohibits, among other things, the take of endangered species. The wildlife agencies routinely apply the prohibitions in section 9 to threatened species. Take is defined broadly to mean harass, harm, pursue, hunt, shoot, wound, kill, trap, capture, or collect, or to attempt to engage in any such conduct.
